# Does the amount of formal care affect informal care? Evidence among over-60s in France

**DOI:** 10.1007/s10198-021-01370-5

**Published:** 2021-09-07

**Authors:** Elsa Perdrix, Quitterie Roquebert

**Affiliations:** 1 Max Planck Institute for Social Law and Social Policy, Munich Center for the Economics of Aging, Munich, Germany; 2 Université de Strasbourg, Université de Lorraine, CNRS, BETA, Strasbourg, France; 3 Université Paris Dauphine - PSL, Leda, Legos, Paris, France

**Keywords:** Long term care, Informal and formal care, Instrumental variable, I10, J14, I18

## Abstract

This paper investigates the causal effect of the amount of formal care used on the informal care received by formal care users. We use an original instrument for formal care volume based on local disparities (NUTS 3 level, 96 units) in the price of formal care. Using the French CARE survey, we use a two-part model to assess the effect of formal care on the extensive and the intensive margin of informal care. An increase in the amount of formal care is found to be associated with a small decrease in the probability of using informal care. Heterogeneity tests show that this negative effect is mainly driven by help for daily activities provided by women. At the intensive margin, informal care is not significantly affected by the amount of formal care. Reforms increasing subsidies for formal care can thus be suspected to have a limited effect on informal care arrangements.

**Supplementary Information:**

The online version contains supplementary material available at 10.1007/s10198-021-01370-5.

## Introduction

Like many European countries, France is having to deal with an aging population and increasing demand for long-term care. Long-term care, i.e. services for individuals with functional limitations, can be provided by professionals (formal care) and non-professionally, by relatives (informal care). Informal care plays a major role in the provision of domestic help and personal care [10], but it has been shown to have detrimental effects on caregivers’ health, labor supply and social life [3]. In France, public policies tend both to encourage the use of professional care services and to support informal caregivers, while long term care insurance is rare. The main program for disabled older adults, the APA (*Allocation personnalisée d’autonomie*), partially subsidizes formal home and personal care (excluding medical expenses) for all individuals older than 60 years with physical limitations, with a subsidy inversely related to the income. In 2018, 1.3 million of adults were receiving the APA (59% living in the community). There are also measures in place to alleviate the burden of informal care for relatives, such as respite solutions (temporary stays in nursing homes). Evaluating such policies requires an understanding of the interactions between formal and informal care.

This paper documents the causal effect of the amount of formal care on informal care provided by relatives to over-60s living at home. Increased formal care use can lead to a decrease in informal care if formal care replaces informal care: care provided formally does not need to be provided by relatives. Conversely, an increase in formal care use may increase the need for informal support, for instance to cope with administrative costs. Formal care may also have a signaling effect, highlighting the importance of the limitations and thereby increasing the level of informal care.

The literature on the effect of informal care provision on formal care use is prolific and typically shows that informal and formal care are substitutes [6]. The impact of formal care on informal care has not been studied to the same extent. Several studies have investigated the impact of public subsidies on both formal and informal care consumption [1, 9, 15, 16, 24, 26, 29], aiming to forecast the effects of public policies financing formal care on care arrangements. They generally find that public subsidies (binary treatment) have a negative effect on informal care. The causal impact of formal care on informal care has seldom been studied. The only published study on this question that we are aware of investigates arrangements in Austria, Belgium, Germany and France [8]. Formal care is instrumented by variations in long-term care program eligibility. These authors find that increased use of formal care may also increase the use of informal care.

We contribute to the literature by analyzing the effect of the amount of formal care on informal care use. To the best of our knowledge, this is the first paper focusing on the effect of formal care intensity on informal care in formal care users. Studying the intensive margin of formal care is of interest in a context where long-term care policies are becoming increasingly important. In France, the 2016 ASV law^1^ increased formal care subsidies for individuals covered by the APA program. Evaluating its effects on informal care requires insight into how increased formal care use affects the amount of informal care received.

We use the 2015 French cross-sectional survey CARE (Capacités, aide et ressources des seniors) and focus on formal care users. We estimate a two-part model, which highlights the effect of formal care both on the extensive and intensive margin of informal care. We use local variations in the price of formal care to deal with endogeneity.

Our results show that an exogenous increase in the volume of formal care has a weak negative effect on the extensive margin of informal care. This negative effect mainly concerns help with everyday activities, provided by women, and it affects both primary (partner and children) and secondary caregivers (friends, neighbours). The intensive margin of informal care is unaffected. Thus, an increase in formal care use such as the one implemented by the 2016 APA reform can be suspected to have a limited effect on the involvement of relatives in the provision of care.

## Conceptual framework

We use the classic theoretical framework generally used in the literature [25]. Here, we present a simplified version of the model in which the utility of the parent (indexed by *p*) and of the child^2^ (indexed by *c*) is denoted $U_i, i \in \{c,p\}$. The child provides a quantity of informal care *IC* while the parent can also consume hours of formal care *FC*. Both contribute to the parent’s wellbeing *W*, and their effect is conditional on the parent’s level of disability *D*. We assume a Cournot–Nash equilibrium where the child unilaterally chooses the quantity of informal care provided, taking the amount of formal care used as given; while the parent unilaterally chooses their formal care use, taking the amount of informal care provided as given.

The child is assumed to be altruistic in caring for their parent. Their utility is expressed as follows:

$$\begin{aligned} \left\{ \begin{array}{l} {\mathrm{Max}}_{ X^{\mathrm{c}}, {\mathrm{IC}}, L} \text { } U^{\mathrm{c}}(X^{\mathrm{c}}, W({\mathrm{IC}}, {\mathrm{FC}}; \,D), L) \\ \text {s/c } V^{\mathrm{c}} + \omega T = X^{\mathrm{c}} + \omega (L + {\mathrm{IC}}) \\ \end{array} \right., \end{aligned}$$

where $V^{\mathrm{c}}$ is the child’s nonlabor income, $\omega$ their labor wage, *T* is the total time endowment, $X^{\mathrm{c}}$ is the consumption of private goods and *L* is leisure.

The parent chooses $X^{\mathrm{p}}$ and FC to maximize their utility:

$$\begin{aligned} \left\{ \begin{array}{l} {\mathrm{Max}}_{X^{\mathrm{p}}, {\mathrm{FC}}} \text { } U^{\mathrm{p}} (X^{\mathrm{p}}, W({\mathrm{IC}}, {\mathrm{FC}};\, D)) \\ \text {s/c } V^{\mathrm{p}} = X^{\mathrm{p}} + p_{\mathrm{FC}} {\mathrm{FC}} \\ \end{array} \right., \end{aligned}$$

where $V^{\mathrm{p}}$ is the parent nonlabor income and $p_{\mathrm{FC}}$ is the price of formal care.

The amount of informal care is chosen by the child while the quantity of formal care used is the parent’s decision. This gives the following reaction functions:^3^

1 $$\begin{aligned}&{\mathrm{IC}}^{\mathrm{c}} = f^{\mathrm{IC}} (V^{\mathrm{c}}, \omega , {\mathrm{FC}}(p_{\mathrm{FC}}); \, D) \end{aligned}$$

2 $$\begin{aligned}&{\mathrm{FC}}^{\mathrm{p}} = f^{\mathrm{FC}} (V^{\mathrm{p}}, p_{\mathrm{FC}}, {\mathrm{IC}}; \, D). \end{aligned}$$

This theoretical framework highlights a possible empirical strategy. Indeed, since in this model, the price of formal care only affects informal care through the formal care function, it may be possible to use the price of formal care as an instrument for formal care use.

## Data and method

### Data and sample

This paper uses two datasets: a national survey of older adults (over-60s) in France and a local-level survey of long-term care practices in France (“*départements*”, NUTS 3, 96 units in Metropolitan France). The latter is used to obtain the instrumental variable.

*The CARE survey* We use the French CARE survey (*Capacités, aide et ressources des seniors—volet ménages*), which focuses on adults over the age of 60 living in the community. This cross-sectional survey, conducted in 2015, surveyed a representative sample of close to 11,000 individuals aged 60 years or older. The survey provides exhaustive information on the limitations faced by individuals and the formal and informal care they receive. This information includes in many cases the number of hours provided by professional caregivers and relatives.

*A local survey* In France, long-term care policies are managed at a local level (NUTS 3) by local authorities called departmental councils.^4^ Our instrument comes from the *SolvAPA* survey [12], conducted in 2015 by the French Ministry of Health to document the long-term care policies of departmental councils.^5^ This survey provides information on how departmental councils manage long-term care and the characteristics of local formal homecare services. Using this survey implies a focus on individuals living in a department that responded to the survey (82 of the 96 metropolitan departments did).

*Sample selection* The study sample consists of individuals living at home who declared using formal care services. We focus specifically on those who use unskilled formal care, provided by professional housekeepers or non-medical caregivers.^6^

Our study population is thus a selective subset of over-60s in France. Indeed, older adults living at home are on average less disabled and more socially integrated than their peers living in nursing homes. Focusing on individuals who use formal care is also selective. Table 1 presents the determinants of formal care consumption for all CARE respondents (Column 1) and among these, on all respondents living in departments that responded to the SolvAPA survey (Column 2). Compared to all adults over the age of 60, formal care users are more likely to be women, live alone, receive APA benefits, and have a low income.^7^ The probability of using formal care increases with age and the level of disability, and is inversely related to the number of children.

Including non-users of formal care would make the sample less selective and would provide results on both the intensive and extensive margins of formal care. However, the resulting sample—including both formal care consumers and non-consumers—is not relevant in our analysis. As shown in Table 1 indeed, the lowest regulated price in the department—the instrument variable—is not correlated with formal care use at the extensive margin: there is no direct link between our instrument and the selection of formal care users. This then suggests that formal care demand is not sensitive to formal care price at the extensive margin.^8^ Our instrument is therefore far less relevant for the extended sample (Appendix B.1).

**Table 1:** Explaining formal care use. Source: CARE survey [13]

	Consumes formal care (probit model)	
	(1)	(2)
Woman	0.449*** (0.0365)	0.446*** (0.0397)
Age	0.0404*** (0.00197)	0.0398*** (0.00210)
Lives alone	0.441*** (0.0383)	0.435*** (0.0415)
Disability group 1	0.224 (0.142)	0.271* (0.156)
Disability group 2	0.378*** (0.0648)	0.353*** (0.0719)
Disability group 3	0.309*** (0.0674)	0.358*** (0.0732)
*Ref: Disability group 4*		
Disability group 5	$-$ 0.383*** (0.0467)	$-$ 0.372*** (0.0473)
Disability group 6	$-$ 1.084*** (0.0392)	$-$ 1.093*** (0.0420)
Has the *baccalauréat*	0.0837** (0.0422)	0.0896* (0.0461)
Has children	$-$ 0.123*** (0.0419)	$-$ 0.121** (0.0483)
Yearly income (/1000)	$-$ 0.00146 (0.00154)	$-$ 0.00135 (0.00161)
Proxy	0.183***(0.0401)	0.182*** (0.0420)
Regulated price (log)		0.0380 (0.246)
Observations	10,290	8,882

Interpretation: Other things being equal, women are more likely to receive formal care than men are. *$\textit{p}<0.10$, **$\textit{p}<0.05$, ***$\textit{p}<0.01$. Standard errors in parentheses. Estimation of probit models among (1) 10,920 respondents to the CARE survey and (2) the 8882 respondents living in a department that responded to the SolvAPA survey

Finally, our sample is restricted to individuals living in departments that responded to the SolvAPA survey^9^ and we exclude outliers, defined as the highest 1% of formal and informal care users.^10^

*Outcome and variable of interest* The variable of interest is the number of hours of formal care received by respondents. The outcome variable is the amount of informal care received, defined as the number of hours of informal care declared.^11^ The amounts of formal and informal care received were directly declared by respondents for each caregiver on a daily, weekly or monthly basis. Since the most frequent unit used was the week, we converted all amounts into weekly hours used. Appendix B.3 presents the distribution of these variables. The distributions are skewed on the original scale but this is corrected by transforming to log-hours. The control variables considered are gender, age, living status (alone or not), number of children, education level (having the French *baccalauréat* or not), income level and disability group. The disability group is a synthetic indicator computed from declared activity limitations and mimicking the AGGIR scale used in the APA program to assess individuals’ disability level.^12^ We also control for proxy respondents.

*Descriptive statistics* Table 2 presents summary statistics for the main variables used in the model for the estimation samples: formal care consumers (Column 1), and, among them, those who receive informal care (Column 2). The baseline sample contains a majority of women and most individuals live alone. They have about two children on average and a moderate level of disability. Informal care users are on average older and have more children. They are more severely disabled, more frequently APA beneficiaries, and responded more often by proxy. As a result, they consume significantly more formal care (7.21 h/week on average, compared with 6.48 h/week in the baseline sample).

**Table 2:** Descriptive statistics for the estimation sample. Source: CARE survey [13]

	Baseline sample		Informal care consumers		Difference between samples
Consumes informal care	56.57	(49.58)	100	(0)	–
Consumes formal care	100	(0)	100	(0)	–
Hours of formal care	6.483	(8.187)	7.207	(9.114)	***
Hours of informal care	13.35	(29.51)	23.60	(36.02)	***
Woman	77.68	(41.65)	78.30	(41.23)	n.s.
Age	82.55	(8.407)	83.85	(8.314)	***
Lives alone	71.03	(45.37)	70.69	(45.53)	n.s.
Number of children	2.341	(1.746)	2.473	(1.780)	***
Has the *baccalauréat*	13.37	(34.04)	10.75	(30.98)	***
APA beneficiary	44.26	(49.68)	48.53	(50.00)	***
Disability group					***
1 (severely disabled)	2.455	(15.48)	3.605	(18.65)	
2	14.24	(34.95)	18.56	(38.89)	
3	13.71	(34.40)	17.36	(37.89)	
4	34.67	(47.60)	33.71	(47.29)	
5	14.46	(35.18)	12.48	(33.06)	
6 (independent)	20.47	(40.35)	14.29	(35.00)	
Yearly income					***
${<}$ €10,000	25.38	(43.53)	24.97	(43.30)	
€10,000–€15,000	27.87	(44.84)	30.91	(46.23)	
€15,000–€20,000	22.05	(41.47)	21.70	(41.23)	
${>}$ €20,000	24.70	(43.13)	22.43	(41.73)	
Proxy	44.07	(49.66)	58.08	(49.36)	***
Observations	2648		1,498		

Interpretation: In the baseline sample (consumers of formal care) 56.57% receive informal care. The average weekly consumption of formal care is 6.48 h. In the sub-sample of informal care consumers, the average weekly consumption of formal care is 7.21 h. The difference between the two samples is significant at the 1% level. Continuous and binary variables were compared using t tests and categorical variables were compared using $\chi ^2$ tests. *$\textit{p}<0.10$, **$\textit{p}<0.05$, ***$\textit{p}<0.01$; n.s, not significant. Standard deviations are in parentheses

### Instrumental variable

We face the classical endogeneity issues that arise when simultaneously studying formal and informal care. The first possible source of endogeneity is reverse causality: what we measured might in fact be the effect of informal care on formal care use. The second is omitted variable bias: unobserved determinants of both formal and informal care use may yield biased estimators.

We deal with these endogeneity problems using an instrumental variable approach. Local variations in home care supply provide an exogenous source of variations in the volume consumed. In particular, we can expect consumption to be higher where prices are lower, since the demand for formal care is sensitive to the price at the intensive margin [28]. Observing prices at the local level rather than at the individual level guarantees that variations in this price are exogenous to individuals’ demand. We consider the departmental level, which is where the home care sector is managed in France [18]. Since we exploit local variations, identification is based on interindividual variations.

The instrument we use is the lowest regulated price available in the department. In France, there are both regulated and non-regulated home care service providers.^13^,^14^ We focus on regulated prices, for which data are available. Regulated providers are allowed to enter the market by departmental councils; they have to meet quality requirements and their prices are set by the departmental council.^15^

The departmental council sets a price for each provider, which depends on their provision costs. It is supposedly set to the average hourly cost of provision but qualitative studies have shown that it mainly depends on administrative considerations [17]. For instance, the departmental council can modulate the proportion of qualified caregivers in the workforce through the pricing process. The heterogeneity in regulated prices thus reflects variations in provision costs as well as departmental variations in pricing practices.

To be valid, our instrument should not affect informal care except through formal care (exclusion restriction). This is what is predicted in the conceptual framework (Eq. 1). This assumption does not hold if informal care is affected by parents’ private goods consumption ($X^{\mathrm{p}}$), which also depends on the price of formal care through parents’ budget constraints [1]. When parents make financial transfers to their children moreover, the price of formal care may affect how much they transfer, and this then modifies the non-labor income of their children ($V^{\mathrm{c}}$), which then affects how much informal care they decide to provide. Strategic behaviors may also come into play: if the cost of formal care is high, children may choose ex ante to increase the amount of informal care they provide to limit their parent’s consumption of formal care and preserve their inheritance. This kind of behavior is probably not widespread since formal care is relatively inexpensive (compared, for instance, to the price of nursing homes). Moreover, this mechanism is only really relevant for relatively-high income individuals.

A problem may arise if individuals can influence regulated prices to suit their existing informal care arrangements. Qualitative studies have shown that the pricing of regulated services is driven by administrative mechanisms [17] and is not identified as a political issue (in the voting process for instance) [4]. Moreover, older adults and their families rarely engage in collective action [31] and are unlikely to influence these technical decisions.

Another problem to consider is the possible correlation between the departmental price and departmental characteristics that reflect informal and formal care uses in the department. In our estimations, we control for a set of departmental variables that correlate with informal care use to limit the effect of any unobserved determinants of informal care. We checked for a correlation between the lowest regulated price and variables related to the characteristics of older adults in the department (share of over-60s in the population, share of women among over-60s, share of over-60s living alone, relative number of nursing home beds, share of APA beneficiaries) and the socioeconomic characteristics of the department (P90/P10 ratio, share of taxable households, poverty rate, political orientation of the departmental council). Table 3 shows that none of these variables are correlated with the lowest regulated price in the department. We also tested whether excluding non-significant variables in a stepwise manner would increase the level of significance of the other associations. The results remained stable. Local characteristics thus explain very little of our instrument’s variability.

**Table 3:** Instrument variations and departmental characteristics. Source: SolvAPA survey [12]

	Lowest regulated price in the department
Share of 75+ in the population (2015)	2.148 (17.39)
Interdecile ratio (2014)	0.720 (0.928)
Share of taxable households (2014)	0.0371 (0.0818)
Share of women among 75+ (2015)	1.571 (32.49)
Share of 75+ living alone (2014)	$-$ 0.0806 (0.189)
Share of 75+ living in nursing home (2014)	0.299 (0.384)
Poverty rate 75+ (2014)	$-$ 0.0628 (0.187)
Equipment rate in institutions: medical beds (2014)	$-$ 0.00958 (0.0376)
Share of APA beneficiaries in the 60+ population (2005)	0.0668 (0.226)
Left-wing departmental council (2015)	$-$ 0.270 (0.570)
Constant	15.28 (17.70)
$R^{2}$	0.087

Interpretation: A one percentage point increase in the share of over-75s in the department is associated with a non-significant increase in the lowest regulated price in the department. Standard errors in parentheses. *$\textit{p}<0.10$, **$\textit{p}<0.05$, ***$\textit{p}<0.01$. Linear regression model among the 76 departments with regulated providers that responded to the SolvAPA survey

**Fig. 1:**
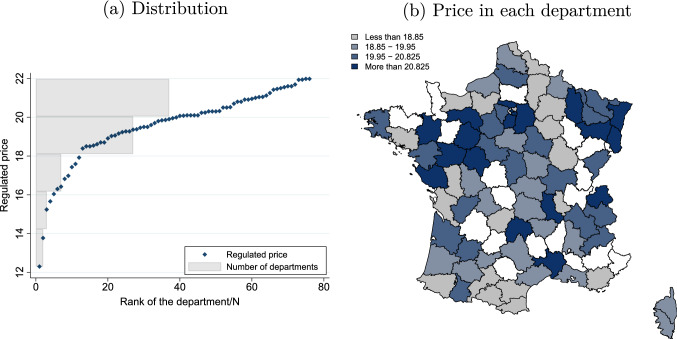
Description of the lowest regulated price. Interpretation: **a**: departments are ranked from high to low by lowest regulated price, which ranges from 22 to 12 euros per hour. 37 departments have a lowest regulated price between 20 and 22 euros per hour. **b** Lowest regulated price in each department shown on a map of France. Missing departments are in white. Source: SolvAPA survey, 2015

In the SolvAPA survey, departmental councils were asked to provide information on the prices set for regulated providers. We consider the lowest price in the department, which indicates the minimum price that has to be paid for formal care from a regulated provider.^16^ In the 76 departments represented in our sample, this price ranges from €12.3 per hour to €21.98 per hour, with an average of €19.54 and a standard deviation of 1.88. Figure 1a shows the distribution of the lowest regulated price. Spatial auto-correlation tests (Appendix D.1) show that there is no correlation between lowest regulated prices in nearby departments. In summary, these tests all indicate that our instrument is exogenous. A final test of the sensitivity of the instrument to exclusion of departments with the lowest regulated price (Appendix D.2) shows that our instrument is not sensitive to the exclusion of extreme values.

None of the alternative instruments investigated, based on local policy or individual characteristics, was found to be as good as the chosen variable (see Appendix D.3 for details).

### A two-part model of informal care use

*Two-part model* We aim to show that there is a causal association between variations in the amount of formal care used and informal care received—both at the extensive and intensive margins. We use a two-part model (TPM) combined with an instrumental variable approach [14],^17^ as has already been used in studies of long term care arrangements [6, 8].

The first part of the model is a binary (probit) model that focuses on the extensive margin of informal care. With the instrumental variable (IV) strategy, this first part involves two stages. The first stage involves calculating the variation in log hours of formal care attributable to the level of the regulated price in the department (Eq. 3) and the second stage involves calculating the variation in the probability of reporting informal care attributable to the exogenous difference in log hours of formal care (Eq. 4). Equation (4) was solved using conditional maximum likelihood estimation.^18^

3 $$\begin{aligned}&{\mathrm{log}}({\mathrm{FC}}_i) = \pi _0 + \pi _1 T_{d(i)} + \pi _2 X_i + \pi _3 Y_{d(i)}+ u_i \end{aligned}$$

4 $$\begin{aligned} {\mathrm{Pr}}({\mathrm{IC}}_i>0 |_{{\mathrm{FC}}_i, X_i,Y_d(i)}) &= \Phi (\alpha _0+ \alpha _1 {\mathrm{log}}({\mathrm{FC}}_i) \nonumber \\& \quad + \alpha _2 X_i + \alpha _3 Y_{d(i)} ) \end{aligned}$$

with ${\mathrm{Pr}}({\rm{IC}}_i>0)$, the probability of individual *i* declaring informal care use; $T_{d(i)}$, the lowest regulated price available in individual *i*’s department *d*; $X_i$, controls for individual characteristics; $Y_{d(i)}$, controls for departmental characteristics. $\Phi$ is the cumulative distribution function of the standard normal distribution. We assume that error terms in Eqs. (3) and (4) are correlated, with a joint normal distribution.

The second part is a two-stage least squares (2SLS) regression explaining the amount of care consumed by informal care users, focusing therefore on the intensive margin of informal care.

5 $$\begin{aligned} {\mathrm{log}}({\mathrm{FC}}_i) &= \tau _0 + T_{d(i)} \tau _1 + X_i \tau _2 \nonumber \\& \quad + Y_{d(i)} \tau _3 + v_{i} , \forall i, IC_i>0 \end{aligned}$$

6 $$\begin{aligned} {\mathrm{log}}({\mathrm{IC}}_i)|_{{\mathrm{IC}}_i>0, {\mathrm{FC}}_i, X_i,Y_d(i)} &= \beta _0 + \beta _1 \widehat{{\mathrm{log}}({\mathrm{FC}}_i}) \nonumber \\& \quad + \beta _2 X_i + \beta _3 Y_{d(i)} + \varepsilon _i \end{aligned}$$

We assume that errors are normally distributed. To account for potential correlations of disturbances between individuals living in the same department, we cluster standard errors at the departmental level [22].

*Covariates* The individual covariates are variables that are likely to be correlated with informal care use: gender, age, living status (alone or not), having children, education level, disability group, proxy respondent and income level.

We also control for relevant department characteristics, namely the share of over-60s in the population, the share of women among over-60s and the availability of nursing home places. We additionally include characteristics related to the political and economic situation of the department, namely the political orientation of the departmental council, the P90/P10 ratio and the local unemployment rate.

## Results

### Main results

Table 4 presents the estimated effect of formal care volume on informal care use and volume. For both the first part and the second part of the model, results are shown for the naive analysis (regressing informal care on formal care directly), the reduced form (estimating informal care directly on the instrument) and the first and second stages of the IV estimation.

The two naive analyses (Table 4, Columns (1) and (5)) predict no significant effect on the probability of consuming informal care. However, this may be because reverse causality or omitted variable bias has canceled out a significant impact of formal care on informal care.

According to the reduced form model, a higher regulated price is associated with a higher probability of receiving informal care (Column (2)). A higher regulated price should be associated with lower formal care consumption and thus a higher probability of consuming informal care. At the intensive margin (Column (6)), in the reduced form model, the regulated price is negatively correlated with the amount of informal care declared by consumers. This negative effect could be a signaling effect (receiving less formal care signals to informal caregivers that the care recipient does not need much help).

*Impact of the regulated price on formal care use* To be a good instrument, the regulated price has to be correlated with individual consumption (relevance condition). In our sample, a 1% higher regulated price is significantly associated at the 1% level with a 0.733% reduction in formal care use (Table 4, Column (3)). This association is also observed for informal care consumers: a 1% higher regulated price is significantly associated at the 5% level with a 0.696% reduction in formal care use (Column (7)). In both cases, the F-statistic is low, indicating that the instrument may be weak.^19^ Confidence intervals calculated (see Appendix E.2) using the conditional likelihood-ratio (CLR) statistic [21], expected to be robust to weak instrument bias in small samples, show that the bias induced by our potentially weak instrument is limited for the first part of our model, but may be more important in the second part of the model.

As a robustness check, we reproduce our main results using the lagged value of the regulated price (see Appendix E.1) obtained from a departmental survey conducted in 2012. Given that different sets of departments responded in 2012 and 2015, the use of the lagged value tests whether our results are robust to an alternative sample (individuals consuming formal care and living in a department that responded to the 2012 survey). Our instrument is stronger in this sample than in the baseline sample and our main results are robust.

*Causal effect of formal care on informal care* At the extensive margin, an exogenous higher amount of formal by one log-hour care consumed is associated with a reduction of 29.4 percentage point (pp) of the probability that individuals declare receiving informal care (Table 4, Column (4)). This effect is significantly different from zero at the 1% level. At the intensive margin (Column (8)), an exogenous higher amount of formal care used does not significantly affect the amount of informal care declared by individuals.

*Size of the effect* A higher amount of formal care by log-hour is equivalent to a 2.718-fold higher amount in formal care use.^20^ This represents 11.13 hours more for an individual with a weekly consumption of 6.48 hours of formal care (the average number of hours consumed in our data).^21^ This higher number of hours of formal care—11.13 h more—is associated with a 29.4 pp reduction in the probability of consuming informal care. Assuming the effect is uniform over the range of the variables, this would mean that increasing the amount of formal care by one hour would reduce the probability of receiving informal care by 2.6 pp^22^. Although significant, the effect of an increase in formal care on the probability of receiving informal care is limited.

**Table 4:** Main results: effect of increased formal care use on informal care. *Source*: CARE survey [13]

	First part				Second part			
	(All)				(Informal care consumers)			
	(1)	(2)	(3)	(4)	(5)	(6)	(7)	(8)
	Naive	RF	1st	IV-Probit	Naive	RF	1**st**	2SLS
	Pr (IC)	Pr (IC)	ln (FC)	Pr (IC)	ln (IC)	ln (IC)	ln (FC)	ln (IC)
Marg. Eff.								
Regulated price (log)		0.382*** (0.146)	$-$ 0.733*** (0.236)			$-$ 1.11** (0.448)	$-$ 0.696** (0.294)	
Formal care hours (log)	$-$ 0.011 (0.00989)			$-$ 0.294*** (0.055)	$-$ 0.0125 (0.032)			1.60 (0.975)
*F*-test	–	–	9.70	–	–	–	5.62	–
$R^2$	–	–	0.22	–	–	–	0.23	–
Individual controls	Yes				Yes			
Departmental controls	Yes				Yes			
Clusters	76				74			
$N$	2648				1498			

Interpretation: In the first stage of the first part (resp. second part), a 1% increase in the regulated price in the department is associated with an average decrease of 0.733% (resp. 0.696%) in the number of hours of formal care consumed per week. An exogenous increase of one log-hour in formal care use is associated with a 29.4 pp decrease in the probability of reporting informal care. Among consumers of informal care, an increase of one log-hour in formal care use is associated with a non-significant decrease in the amount of informal care received.

*Marg. Eff.* marginal effect, *RF* reduced form, *1st* first stage

*$\textit{p}<0.10$, **$\textit{p}<0.05$, ***$\textit{p}<0.01$. Standard errors in parentheses, clustered at the departmental level. Individuals and departmental characteristics are controlled for. The regulated price is the lowest regulated price available in the department. Models of Eqs. 3, 4, 5, 6

*Underlying mechanisms* The negative effect we find at the extensive margin may reflect a limited replacement of informal care by formal care: when more care is provided formally, some relatives may stop providing informal care. Since the information on care comes from a declarative survey, the effect may also reflect a declarative bias: care recipients who use more formal care may be less likely to recognize informal care as such. Receiving visits from relatives may not be directly associated with care provision since this is already provided by paid formal caregivers.

### Extensions: alternative outcomes

The richness of the data on caregivers and the type of care they provide makes it possible to explore heterogeneity in the effect of formal care use on informal care. We consider heterogeneity in terms of the kind of care provided (for daily activities, moral support, material help) and the characteristics of the caregiver (relationship with the individual, gender). These heterogeneity tests focus on the first part of the model.^23^ We additionally explore, among the over-60s that reported receiving informal care, an alternative measure of informal care by quantifying it in terms of the number of caregivers reported.

*Type of care* There is evidence in the literature that how substitutable formal and informal care are depends on the type of care [6]. Respondents to the CARE survey were invited to specify what type of care each of their declared caregivers provided: in our sample, among informal care recipients, 99.73% reported receiving help for daily activities, 54.81% moral support and 8.80% material support. Table 5 shows the effect of a higher volume of formal care on the probability of receiving these three types of care (Columns 1–3). A higher volume of formal care is significantly associated with a lower probability of receiving informal care for daily activities. This echoes our main result since almost all declared informal care is associated with everyday activities. However, receiving more formal care does not affect the probability of informally receiving moral support or material help. Thus, the effect of formal care on informal care at the extensive margin is concentrated in daily activities, which can be performed by both types of care providers, but is absent in the types of care that are mostly provided by informal caregivers.

*Relationship with the care recipient* The effect of a change in the amount of formal care used may depend on the caregiver’s characteristics, especially their relationship with the care recipient. In our sample, among informal care recipients, 84.65% reported receiving care from their partner or a child (hereafter referred to as “primary caregivers”)^24^ and 25.10% from neighbors, friends or other family members (“secondary caregivers”). A higher level of formal care received reduces the probability of reporting informal care from both primary and secondary caregivers (Table 5, Columns 4 and 5). For primary caregivers however, the effect vanishes when partners and children are considered separately (Columns 6 and 7). The result for secondary caregivers echoes previous findings that friends and neighbors often stop providing informal care when formal care is also supplied [9].

*Caregiver gender* We are also interested in the interaction between the effect of formal care intensity and caregiver gender. In our sample, 66.56% of respondents reported receiving care from at least one woman and 53.47% from at least one man. A higher amount of formal care use is associated with a significantly lower probability of reporting informal care from women, but not from men (Table 5, Columns 8 and 9). This result echoes differences in the tasks performed by men and women [5], with women providing help with domestic activities and personal care and men being more likely to provide material or administrative help. Formal care is more readily replaced in the tasks performed by women than in those performed by men.

**Table 5:** Extensions: care and caregiver characteristics. *Source*: CARE survey [13]

	Probability to receive informal care								
	Marginal effects								
	Daily life activities	Moral support	Material help	Primary caregivers	Secondary caregivers	Partner	Children	Women	Men
Formal care hours (log)	$-$ 0.250*** (0.065)	$-$ 0.067 (0.119)	$-$ 0.064 (0.096)	$-$ 0.238*** (0.103)	$-$ 0.161** (0.079)	$-$ 0.153 (0.123)	$-$ 0.184 (0.120)	$-$ 0.225** (0.088)	$-$ 0.119 (0.152)
Observations	2648	2648	2648	2648	2648	2648	2648	2648	2648

Interpretation: An increase of one log-hour in formal care use is associated with a 25.0% point increase in the probability of receiving informal care for daily activities. *$\textit{p}<0.10$, **$\textit{p}<0.05$, ***$\textit{p}<0.01$. Standard errors in parentheses, clustered at the departmental level. Individuals and departmental characteristics are controlled for. Formal care hours are instrumented by the lowest regulated price available in the department. Estimation of IV-probit models, marginal effects reported

*Number of caregivers* Our main results show that there is an effect at the extensive margin of informal care but not at the intensive margin. We now consider an alternative measure of the quantity of informal care by counting the number of caregivers (rather than the number of hours of care received). In our sample, among recipients of informal care, the average number of caregivers was 1.77. 57% reported a single caregiver, 24.37% two caregivers and 17.75% reported three caregivers or more. Quantifying informal care in this way does not affect our conclusion at the intensive margin of informal care: the amount of formal care received is not significantly associated with the reported number of informal caregivers.^25^

### Extensions: results for an alternative sample and subsamples

*Effect among benefit (APA) recipients* The effect of formal care on informal care might depend on the disability level of the recipient. Results for the subsample of individuals receiving APA benefits (Table 6) are similar to the baseline results: a higher amount of formal care use is associated with a lower probability of declaring informal care, but does not affect informal care at the intensive margin. Our instrument is stronger than in the baseline sample, suggesting that the regulated price is particularly relevant to explain the formal care consumption of APA beneficiaries. This stems from the fact that most APA beneficiaries (75%) are required to use regulated care providers [11]. In Appendix B.1, Table B2 shows similar results for the subsamples of respondents with limitations in instrumental activities of daily living (IADL) and limitations in activities of daily living (ADL).

*Effect among those living alone* Table 7 shows the results for the subsample of individuals living alone. According to the first part of the analysis, an log-hour higher amount of formal care use is associated with a 27.1 pp lower probability of reporting informal care. This is similar to our baseline results. The second part cannot be interpreted because the association in the first stage is barely significant and the F-statistic is very low. This change in the results for the first stage could be explained by individuals living alone being less sensitive to prices, possibly because they are more disabled.

**Table 6:** Effect of the amount of formal care on informal care use for APA beneficiaries. Source: CARE survey [13]

	First part		Second part	
	(All)		(Informal care > 0)	
	(1)	(2)	(3)	(4)
	1st	IV-Probit	1st	2SLS
	ln (FC)	Pr (IC)	ln (FC)	ln (IC)
Marg. Eff.				
Regulated price (log)	$-$ 0.794*** (0.231)		$-$ 1.054*** (0.313)	
Formal care hours (log)		$-$ 0.325*** (0.039)		0.913 (0.163)
*F*-test	11.74		11.31	
$R^{2}$	0.14		0.16	
Clusters	75		72	
$N$	1172		727	
Individual controls	Yes		Yes	
Departmental controls	Yes		Yes	

Interpretation: In the first stage of the first part (resp. second part), a 1% increase in the regulated price in the department is associated with an average decrease of 0.793% (resp. 1.054%) in the number of hours of formal care consumed per week. An exogenous increase of one log-hour in formal care use is associated with a 32.5 pp decrease in the probability of reporting informal care. Among consumers of informal care, an increase of one log-hour in formal care use is associated with a non-significant increase in the amount of informal care received. *$\textit{p}<0.10$, **$\textit{p}<0.05$, ***$\textit{p}<0.01$. Standard errors in parentheses, clustered at the departmental level. The regulated price is the lowest regulated price available in the department. Models of Eqs. (3), (4), (5), (6)

*Marg. Eff.* marginal effect, *RF* reduced form, *1st* first stage

**Table 7:** Effect of the amount of formal care on informal care use for individuals living alone. *Source*: CARE survey [13]

	First part		Second part	
	(All)		(Informal care > 0)	
	(1)	(2)	(3)	(4)
	1st	IV-probit	1st	2SLS
	ln (FC)	Pr (IC)	ln (FC|IC $$>0)$$	ln (IC)
Marg. eff.				
Regulated price (log)	$-$ 0.770*** (0.221)		$-$ 0.492* (0.254)	
Formal care hours (log)		$-$ 0.271*** (0.059)		1.97 (1.511)
*F*-test	12.14	–	3.76	–
$R^{2}$	0.24	–	0.26	–
Individual controls	Yes		Yes	
Departmental controls	Yes		Yes	
Clusters	76		74	
$N$	1,881		1,059	

Interpretation: In the first stage of the first part, a 1% increase in the regulated price in the department is associated with an average decrease of 0.770% in the number of hours of formal care consumed per week. An increase of one log-hour in formal care use is associated with a 27.1 pp decrease in the probability of reporting informal care. Among informal care consumers, an exogenous increase of one log-hour in formal care use is associated with a non-significant decrease in the amount of informal care received. *$\textit{p}<0.10$, **$\textit{p}<0.05$, ***$\textit{p}<0.01$. Standard errors in parentheses, clustered at the departmental level. The regulated price is the lowest regulated price available in the department. Models of Eqs. (3), (4), (5), (6)

*Marg. Eff.* marginal effect, *RF* reduced form, *1st* first stage

## Discussion

This paper highlights the impact of an exogenous variation in amounts of formal care used on whether and how much informal care is received by formal care recipients. There are several points of discussion regarding the empirical strategy, the data and comparisons with the existing literature.

*Co-residence* A higher level of formal care use might affect the likelihood of cohabitation. In our sample, the correlation between the lowest regulated price and co-residence with children is close to zero (0.008), suggesting that our instrument is hardly if at all related to the probability of living with a child. We found no association between increased formal care use, instrumented by the lowest regulated price, and the probability of living with a child.^26^

*Quantified informal care* The outcome variables we consider are the probability of declaring receiving informal care and the amount of care received. For the first and second parts of our model to be consistent, informal care recipients are defined as respondents who quantified the number of hours of informal care they received. In our sample however, 17% of individuals declared receiving informal care but did not report the number of hours received. Repeating the first part of our analysis, using as outcome variable a dummy indicating whether or not respondents declared any informal care, quantified or not, (Appendix E.4) shows that our results are robust to this change of definition.

*Comparison with previous results* Our results can be compared to those of three recent studies [1, 2, 8].^27^ Using SHARE data, Carrino et al. [8] estimate the causal effect of formal care on informal care use, finding that the amount of formal care has a positive effect on both the extensive and intensive margins of informal care. This difference with our results has several possible explanations. First, since Carrino et al. consider both consumers and non-consumers of formal care, they capture the effects of a change at both the extensive and the intensive margin of formal care. Moreover, their sample covers several European countries (while ours is restricted to France), and a earlier time period: our data are from 2015 while they use the SHARE waves from 2004 and 2006, at the very beginning of the APA policy in France. In their analysis based on the French *Handicap-Santé Ménages* survey (2008), Arnault & Goltz (2014), use out-of-pocket expenses for formal care as an instrumental variable in a bivariate Tobit model [2]. They show that an exogenous increase in formal care use is associated with lower informal care use. Although their study population differs from ours,^28^ the findings of the two studies are consistent and indicate that the decrease is concentrated at the extensive margin of informal care. Arnault [1], using the same data, avoids the assumptions of the IV hypothesis and estimates a reduced-form model identifying the cross-price elasticity of formal and informal care volumes. He finds that more expensive formal care in a department^29^ is associated with a reduction in formal care consumption but not in informal care use. The differences in the results obtained in different populations, and the different ways formal and informal care can be measured, highlight the complexity of the relationship between the two types of care. Further investigations accounting for the heterogeneity in long-term care recipients are required.

This paper contributes to the literature on long-term care arrangements by highlighting the effect of the amount of formal care on informal care. Limitations and perspectives for future research are the following. First, our data are cross-sectional. While it comes with a rich information on care provided to the individuals, longitudinal data would be useful to reinforce the causal aspect of the analysis. Second, our paper is one of the first to propose an instrument for formal care use and it is based on local variations in the long-term care sector. It is then necessary to assume that these local variations do not affect informal care otherwise than their effect on formal care consumption. While we have reasons to think this assumption holds, future investigations should explore alternative instruments to compare results and refine the analysis. Finally, our results are specifically focused on formal care consumers and we only analyze the intensive margin of formal care. Further research should explore and identify separately the causal effect of the intensive and extensive margin of formal care on informal care.

## Conclusion

This paper documents the effect of an exogenous variation in formal care, among formal care consumers, on informal care, both at the intensive and the extensive margin. This effect is estimated for formal care users only. To avoid endogeneity, we use an original instrumental variable approach based on local disparities in the price of regulated providers. Using a two part model, we show that an increase in formal care use is associated with a significant but near-zero decrease in the probability of reporting informal care use. Heterogeneity tests show that this negative effect mainly concerns help with daily activities, provided by women, and affects both primary and secondary caregivers. At the intensive margin of informal care, however, no significant effect is observed. Overall, informal care arrangements can be expected to remain relatively stable even if subsidies for formal care are increased.

## Supplementary Information

Below is the link to the electronic supplementary material.

Supplementary file1 (PDF 881 kb)

## Footnotes

1 *Loi relative à l’adaptation de la société au vieillissement*—law for the adaptation of society to aging.

2 Since we are not interested in long term care arrangements within the family, we only consider the total volume of informal care, however many carers are involved. We therefore aggregate all the informal care provided into a single child, whatever the actual number of caregivers.

3 These functions come from the first order condition of the utility function. See Appendix A for details and [25] for further details on the resolution of this maximization program.

4 *“Conseils départementaux”*.

5 The questionnaire is available online (in French): http://www.data.drees.sante.gouv.fr/TableViewer/document.aspx?ReportId=344.

6 In the CARE survey, these professionals are referred to as “aide à domicile”, “auxiliaire de vie”, “garde à domicile”, “femme de ménage”, and “aide-ménagère”.

7 This effect of income has already been observed in French data [27]. This may be because even when disability status is controlled for, income is associated with unobserved dimensions of health, such that low income reflects higher care needs.

8 Several papers dealing with the price sensitivity of formal care demand have shown that demand is sensitive to the price at the *intensive margin* [26, 28]. At the extensive margin, results are less convincing. A study in Canada found a positive association between the level of subsidies and formal care use, which was not significant however when the level of subsidies was instrumented [29]. In France, receiving public subsidies is associated with a higher probability of receiving formal care but this effect is weak for highly disabled individuals [16]. In France moreover, more generous local benefits are not associated with greater use of formal care [27]. The evidence is thus mixed and suggests that the price sensitivity of formal care use at the extensive margin is heterogeneous and depends on individuals’ level of disability.

9 76 departments of the 82 that responded are represented in the sample. The characteristics of formal care users from departments that did not respond to the *SolvAPA* survey (420 observations) do not differ significantly from those of formal care users in general.

10 This corresponds to a threshold of more than 167 hours of informal care per week or more than 70 hours of formal care per week. Appendix B.2 presents alternative estimations with different outlier definitions.

11 Respondents were only considered recipients of informal care if they had declared the number of hours provided by at least one of their caregivers. More details are provided in Appendix E.4. Our results are robust to an alternative definition of informal care reception.

12 More details on the contents of each category are given in Appendix C.

13 Prior to a 2016 reform making regulation mandatory in the home care sector, service providers could be regulated or not. Our data were collected in 2015, when this distinction was still relevant

14 A previous study describes in detail the different types of home care service providers in France [27].

15 In contrast, non-regulated providers are free to enter the market and are only limited in their rate of price increase.

16 Individuals could potentially purchase services at lower prices from non-regulated providers—informal workers in particular. No data are available, however, on these prices. Moreover, departmental councils tend to encourage the use of regulated providers for individuals receiving APA benefits: most beneficiaries (75%) are required to use a regulated provider [11].

17 See [20] for a review of econometric tools for healthcare resources and costs and long term care consumption studies Mihaylova et al. [20]; see Leung and Yu [19] for a comparison of sample selection and two part models.

18 We used the Stata command “ivprobit” (version 14.0), which uses either maximum-likelihood estimation (MLE) or Newey’s efficient two-step estimator [23]. Newey’s estimator is useful when MLE does not converge but does not allow clustering of standard errors. Since there are no problems of convergence in the main analysis, we use MLE estimation and cluster standard errors at the departmental level (see below).

19 The usual rule of thumb states that the instrument is weak when the F-statistic is lower than 10 [30]. It applies to the case of IID errors and is thus not relevant to our estimation with clusters [7]. When clusters are not included, the *F*-statistic for our baseline results is 14.77 for the first part and 7.04 for the second part (available upon request).

20 $${\rm{ln}} x_2 = ln x_1 + 1 \Leftrightarrow ln \frac{x_2}{x_1} = 1 \Leftrightarrow \frac{x_2}{x_1} = e^{1} = 2.718 \Leftrightarrow x_2 = 2.718 \times x_1$$

21 $$(2.718 \times 6.48)- 6.48.$$

22 $$\frac{0.294}{11.13} = 0.026.$$

23 The intensive margin cannot be studied for some variables (number of hours not declared for all types of care) and for others, the small number of consumers in the second part often undermine the validity of the first stage.

24 22.90% from their partner, 66.36% from children.

25 The corresponding regression coefficient is 0.367 with a standard error of 0.564. More details available opon request.

26 Results available upon request. The marginal effect of formal care volume on the probability of co-residence is 0.088, with a robust standard error of 0.120.

27 Chapter 4 of Arnault’s thesis [1]

28 Arnault and Goltz [2] focus on older individuals living alone with ADL and IADL.

29 This variable corresponds to the price of the largest provider in the department.
