# Supplementary material for: Does the amount of formal care affect informal care? Evidence among over-60s in France
Source: Eur J Health Econ. 2021 Sep 7;23(3):453–65. doi: 10.1007/s10198-021-01370-5 (PMC8964663; doi:10.1007/s10198-021-01370-5)
Supplement: Supplementary file 1 — Supplementary file1 (PDF 881 kb) [file 10198_2021_1370_MOESM1_ESM.pdf]

Does the amount of formal care affect informal care? Evidence among over-60s in France

Elsa Perdrix Quitterie Roquebert

Appendix

A Details on the theoretical framework

The child is assumed to be altruistic in considering the wellbeing of their parent. The child's utility is formalized as follows:

$$\begin{cases} Max_{X^c,IC,L}\ U^c\Big(X^c, W(IC, FC; D), L\Big) \\ \text{s/c } V^c + \omega T = X^c + \omega(L + IC) \end{cases} \tag{1}$$

With V^c the child's non-labor income, ω their labor wage, T the total time endowment, X^c the consumption of private goods and L leisure.

$$Equation\ 1 \Rightarrow Max_{X^c,IC}\ U^c\Big(X^c, W(IC, FC; D), L(IC, X^c, V^c)\Big) \tag{2}$$

Considering a Cournot-Nash equilibrium, the child chooses the optimal amount of informal care, taking the amount of formal care as given. Thus,

$$Equation\ 2 \Rightarrow Max_{X^c,IC}\ U^c\Big(X^c, W(IC, \bar{FC}; D), L(IC, X^c, V^c)\Big) \tag{3}$$

The first order condition is:

$$\begin{cases} \frac{\partial U^c\left(X^c, W(IC, FC; D), L\right)}{\partial X^c} = 0 \\ \frac{\partial U^c\left(X^c, W(IC, FC; D), L\right)}{\partial IC} = 0 \end{cases} \tag{4}$$

The parent chooses X^p and FC to maximize their utility:

$$\begin{cases} Max_{X^p, FC}\ U^p\Big(X^p, W(IC, FC; D)\Big) \\ \text{s/c } V^p = X^p + p_{FC}FC \end{cases} \tag{5}$$

Where V^p is the parent's non-labor income and p_{FC} is the price of formal care.

$$Equation\ 5 \Rightarrow Max_{X^p, FC}\ U^p\Big(X^p(V^p, FC), W(IC, FC(V^p, X^p; D)\Big) \tag{6}$$

Considering a Cournot-Nash equilibrium, the parent chooses the optimal amount of formal care, taking the amount of informal care as given. Thus, the first order condition is:

$$\begin{cases} \frac{\partial U^p\left(X^p(V^p, FC), W(IC, FC(V^p, X^p; D)\right)}{\partial X^p} = 0 \\ \frac{\partial U^p\left(X^p(V^p, FC), W(IC, FC(V^p, X^p; D)\right)}{\partial FC} = 0 \end{cases} \tag{7}$$

First order conditions give the following reaction functions:

$$IC^c = f^{IC}(V^c, \omega, FC(p_{FC}); D) \tag{8}$$

$$FC^p = f^{FC}(V^p, p_{FC}, IC; D) \tag{9}$$

B Detailed description of the sample

B.1 Sample of older adults and subsample of formal care consumers

Our main results concern over-60s receiving formal care. Table B1 and Table B2 present the results for an extended sample, including both consumers and non-consumers of formal care. Since we log-transform the formal care variable, values are increased by 1 to avoid zeros. In each group, we distinguish individuals by disability levels, using limitations in instrumental activities of daily living (IADL) and limitations in essential activities of daily living (ADL). Respondents were regarded as having IADL or ADL limitations if they declared having some or a lot of difficulty in performing an activity, or if they needed assistance to perform it. Table B1 presents the first stage and Table B2 the second stage of the analysis (both at the extensive and intensive margin of formal care).

Table B1 presents the first stage for both the first part of the model (extensive margin, lines 1 to 3) and the second part of the model (intensive margin, lines 4 to 6). When considering the enlarged sample (all respondents, regardless of their care consumption and type of limitation), the regulated price is weakly correlated with the number of hours of formal care and the instrument is weak. The correlation between the regulated price and the number of hours of formal care consumed is higher (with a higher F statistic) in the restricted sample of formal care users. In both cases, smaller samples are associated with a lower F statistic.

Table B2 shows the second stage estimates for the same samples. When all respondents are considered (including those who do not consume formal care), an increase in formal care use is associated with a decrease in the probability of consuming informal care. The results focusing on formal care users are similar. There are no significant effects overall in the second part of the model.

Note that to make all the results in these tables comparable, formal care was quantified in log hours plus one. The results are therefore not directly comparable with our main results, for which the formal care variable was also log-transformed but without adding

one, since non-consumers were excluded.

Table B1: Impact of the regulated price on formal care use with different samples

	All individuals			Formal care consumers		
	All	IADL limitation	ADL limitation	FC consumers	IADL limitation	ADL limitation
**Dependent variable: formal care hours**						
**All individuals**						
Regulated price (log)	-0.156*	-0.254*	-0.251	-0.564***	-0.578***	-0.572**
	(0.0832)	(0.148)	(0.209)	(0.188)	(0.190)	(0.217)
F-test	3.50	2.96	1.44	9.00	9.28	6.93
N	8,882	5,402	3,420	2,648	2,600	2,001
**Dependent variable: formal care hours**						
**Informal care consumers**						
Regulated price (log)	-0.346*	-0.356*	-0.298	-0.550**	-0.551**	-0.536*
	(0.202)	(0.214)	(0.259)	(0.238)	(0.243)	(0.277)
F-test	2.94	2.78	1.39	5.33	5.14	3.73
N	2,966	2,295	2,050	1,498	1,492	1,237

Notes: * $p < 0.10$, ** $p < 0.05$, *** $p < 0.01$. Standard errors in parentheses, clustered at the departmental level. Individuals and departmental characteristics are controlled for. Estimation of linear models.
Source: CARE survey [6].

Table B2: Impact of formal care use on informal care with different samples

	All individuals			Formal care consumers		
	All	IADL limitation	ADL limitation	FC consumers	IADL limitation	ADL limitation
**Dependent variable: probability to consume informal care (Marg. Eff.)**						
Formal care hours	-0.410***	-0.316***	-0.285***	-0.375***	-0.368***	-0.369***
	(0.0229)	(0.0161)	(0.00887)	(0.0677)	(0.0678)	(0.0533)
N	8,882	5,402	3,420	2,648	2,600	2,001
**Dependent variable: volume of informal care hours among consumers**						
Formal care hours	1.963	1.830	1.988	2.025	2.163*	1.898
	(1.351)	(1.428)	(2.348)	(1.273)	(1.290)	(1.497)
N	2,966	2,846	2,050	1,498	1,492	1,237

Notes: * $p < 0.10$, ** $p < 0.05$, *** $p < 0.01$. Standard errors in parentheses, clustered at the departmental level. Individuals and departmental characteristics are controlled for. Formal care volume is instrumented using the lowest regulated price of the department.
Source: CARE survey [6].

B.2 Exclusion of extreme values in the sample

We tested whether our results are sensitive to the exclusion of extreme values in the sample (Table B3). In our baseline analysis, respondents with extreme values of formal and informal care consumption (beyond the 99th percentile)[1] are excluded from the sample. With no exclusion or with a 5% exclusion threshold,[2] the association in the second part of

[1] Corresponding to more than 167 hours of informal care per week or more than 70 hours of formal care per week.

[2] Corresponding to more than 24 hours of informal care per week or more than 84 hours of formal care per week.

the model becomes significant. Our results for the intensive margin of informal care are therefore sensitive to the exclusion of individuals and should thus be interpreted cautiously.

Table B3: Sensitivity tests for outlier exclusion

	First part (All)		Second part (Informal care consumers)	
	(1)	(2)	(3)	(4)
	Regression	IV-Probit	Regression	IV-regression
	$ln(FC)$	$Pr(IC > 0)$ Marg. Eff.	$ln(FC \mid IC > 0)$	$ln(IC \mid IC > 0)$
**No exclusion**				
Regulated price (log)	-0.740***		-0.743***	
	(0.239)		(0.273)	
Formal care hours (log)		-0.279***		1.739**
		(0.056)		(0.958)
F-test	9.63	-	7.40	-
R^2	0.22	-	0.24	-
N	2,689		1,529	
**Exclusion of extreme 5% of formal consumers**				
Regulated price (log)	-0.623***		-0.542***	
	(0.191)		(0.232)	
Formal care hours (log)		-0.335***		2.139*
		(0.290)		(1.298)
F-test	10.60	-	5.42	-
R^2	0.25	-	0.20	-
N	2,453		1,332	

Interpretation: Using our sample without excluding formal and informal care use outliers, a 1% increase in the regulated price in the department is associated with an average decrease of 0.740% in the number of hours of formal care used per week (first stage). An exogenous increase of one log-hour in the amount of formal care consumed is associated with a 27.9 percentage point decrease in the probability of reporting informal (second stage). Among informal care consumers, a 1% increase in the regulated price in the department is associated with an average decrease of 0.743% in the number of formal care hours consumed per week (first stage).
Notes: * $p < 0.10$, ** $p < 0.05$, *** $p < 0.01$. Standard errors in parentheses, clustered at the departmental level. Individuals and departmental characteristics are controlled for. The regulated price is the lowest regulated price available in the department. Models of Equations **??**, **??**, **??**, **??**. Marg. Eff., marginal effect; RF, reduced form; 1st, first stage.
Source: CARE survey [6].

B.3 Distribution of formal and informal care variables

Distributions of the number of hours of care in our main sample and in the sub-sample of informal care consumers (Figure B1) have a first moment of zero and a long right tail. After log transformation (Figures B2), both distributions are close to normal.

Figure B1: Weekly amounts of formal and informal care (in hours)

(a) Formal care distribution

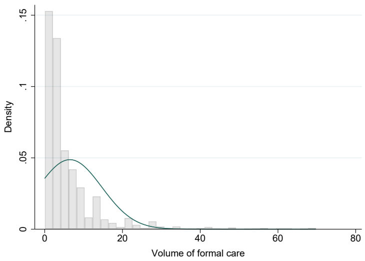

(b) Informal care distribution

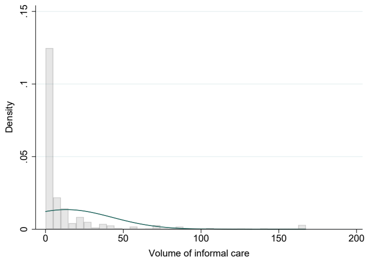

(c) Formal care distribution among formal care consumers

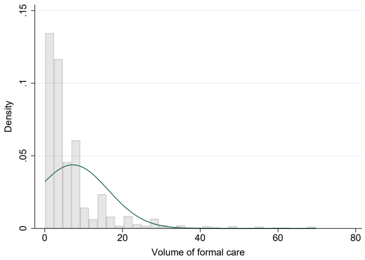

(d) Informal care distribution among formal care consumers

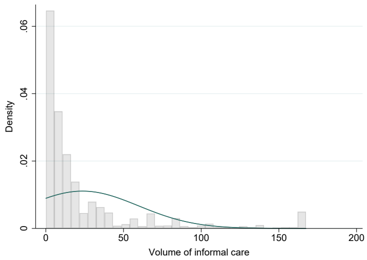

Notes: Distribution of the number of hours of care in our baseline sample and among formal care consumers.
Source: CARE survey [6].

Figure B2: Weekly amounts of formal and informal care (in log hours)

(a) Formal care distribution

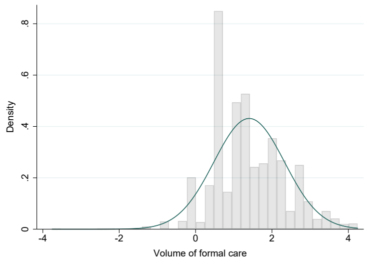

(b) Informal care distribution

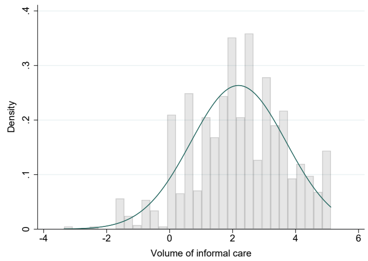

(c) Formal care distribution among formal care consumers

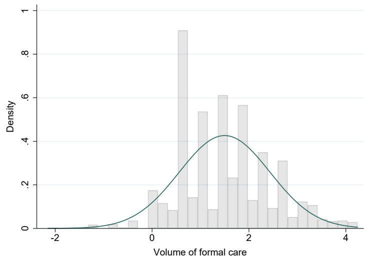

(d) Informal care distribution among formal care consumers

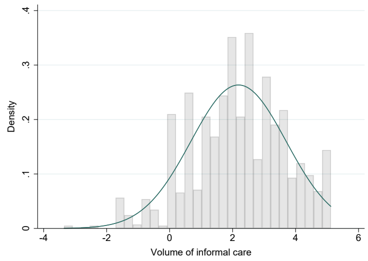

Notes: Distribution of the number of log hours of care in our baseline sample and among formal care consumers.
Source: CARE survey [6] [6].

C Description of support programs for older adults in France

Since 2002, disabled over-60s in France have been entitled to benefits from a specific program, dubbed personalized autonomy allowance (*Allocation personnalisée d'autonomie*, APA). This program subsidizes formal care for over-60s with a disability. This is a government-run, national program, which is delivered at the local level by departmental councils.

Eligibility is conditioned on age (60 years or older) and a disability assessment. Applicants are assessed at home by medical social workers from the departmental council using the French AGGIR (*autonomie, gérontologie, groupe iso ressource*) scale. Individuals are classified in six disability levels, from GIR 1 (severely disabled) to GIR 6 (no limitations). Table C1 summarizes the classification and the disabilities associated with each group. All individuals in the same group are supposed to require the same amount of resources to cope with their activity limitations. Thus, each group is assumed to have the same long term care needs. Only individuals in GIR 1 to 4 are eligible to receive the APA.

For eligible individuals, social workers estimate the number of care hours, referred to as a “care plan" (*plan d'aide*), required to allow them to perform activities of daily living. The monetary value of this care plan must not exceed a given GIR-specific threshold established at the national level. The APA beneficiary is then free to consume the number of care hours they want at a subsidized rate, with copayments increasing with income.

The 2016 reform extended subsidies in two ways. First, the method of calculating the copayment rate was changed so that it now depends on income and disability level, leading to lower copayment rates, especially for low-income and/or severely-disabled individuals. Second, the level of the national GIR-specific ceilings were increased, increasing the size of care plans overall.

Table C1: Definition of disability levels (AGGIR scale)

GIR 1	severely disabled (bedridden) individuals with severe cognitive disorders, in need of constant care
GIR 2	severely disabled individuals with less severe cognitive disorders than those in GIR 1, or able-bodied individuals with severe cognitive disorders
GIR 3	individuals requiring everyday help for personal care but without cognitive impairment
GIR 4	individuals requiring assistance, either getting out of bed, preparing meals, dressing or undressing
GIR 5	individuals needing help from time to time with daily activities, but not on a regular basis
GIR 6	no daily activity limitations

D Detailed description of the instrument

D.1 Spatial autocorrelation

To further document the sources of variation in our instrument, we investigated spatial autocorrelation using Moran's index [10]. In this case, Moran's I measures the extent to which nearby departments have closer levels of regulated price than others. It is computed as follows:

$$I = \frac{N}{S_0} \frac{\sum_i \sum_j w_{i,j}(y_i - \bar{y})(y_j - \bar{y})}{\sum_i (y_i - \bar{y})^2}$$

with N the total number of departments, y_i the lowest regulated price in department i; $\bar{y}$ the average price computed over all available departments, and $S_0 = \sum_i \sum_j w_{i,j}$, $w_{i,j}$ the spatial weights for departments i and j.

The spatial weights we used are the distances between department centers, such that the importance of other departments are weighted in terms of their proximity. This also takes the size of the department into account.[3]

Moran's I with this spatial weighting is a measure of the auto-correlation between the distance between departments and the lowest regulated price of the departments. The value of the index is $I = -0.00699$, with a p-value of 0.365, indicating that there is no spatial correlation between the lowest regulated price in a department and its location with respect to others.

D.2 Exclusion of departments with particularly low regulated prices

We test the robustness of our results to the exclusion of departments that are outliers in terms of the lowest regulated price (Table D1). The values of the F statistic for the first stages of the analysis are lower when the most extreme and two most extreme departments in this regard are excluded. Results for the second stage of the first part of the analysis

[3] An alternative approach would have been to only consider neighboring departments, but this is limiting because for instance, it would not have captured the similarities of non-neighboring departments in the same region.

are consistent with our baseline results. In the second stage of the second part of the analysis, the association between the amounts of formal and informal care is positive and statistically significant at the 10% level. Our results on the intensive margin of informal care are sensitive to the exclusion of departments and should therefore be interpreted cautiously.

Table D1: Sensitivity of results to the exclusion of departments with particularly low regulated prices

	**First part (All)**		**Second part (Informal care consumers)**	
	(1)	(2)	(3)	(4)
	Regression	IV-Probit	Regression	IV-regression
	$ln(FC)$	$Pr(IC > 0)$ Marg. Eff.	$ln(FC \mid IC > 0)$	$ln(IC \mid IC > 0)$
**Exclusion of the department with the smallest price**				
Regulated price (log)	-0.652**		-0.603*	
	(0.251)		(0.326)	
Log of total hours of formal care		-0.321***		2.349*
		(0.0324)		(1.426)
F-test	6.77		3.42	
R^2	0.22		0.28	
Observations	2,636	2,636	1,489	1,489
**Exclusion of the two departments with the smallest prices**				
Regulated price (log)	-0.642**		-0.686**	
	(0.278)		(0.354)	
Log of total hours of formal care		-0.321***		2.520*
		(0.0360)		(1.432)
F-test	5.35		3.76	
R^2	0.22		0.23	
Observations	2,619	2,619	1,481	1,481

Interpretation: In the first part (resp. second part), a 1% increase in the regulated price in the department is associated with an average decrease of 0.652% (resp. 0.603) in the number of hours of formal care consumed per week. An exogenous increase of one log-hour in the amount of formal care consumed is associated with a 32.1 percentage point decrease in the probability of reporting informal care. The first part of the Table corresponds to the estimation excluding the 12 individuals living in the department with the lowest regulated price and the second part to the estimation excluding a further 17 individuals from the department with the second lowest price.
Notes: * $p < 0.10$, ** $p < 0.05$, *** $p < 0.01$. Standard errors in parentheses, clustered at the departmental level. Individuals and departmental characteristics are controlled for. The regulated price is the lowest regulated price available in the department. Models of Equations **??**, **??**, **??**, **??**.
Source: CARE survey [6].

D.3 Finding an instrumental variable for formal care studies

Instrumental variables for formal care are relatively scarce in the literature. Using the SHARE data in Austria, Belgium, Germany and France, [4] used inter-regional variations

in eligibility rules for formal care subsidies. This instrument requires specific assumptions about the sources of inter-regional variations and is difficult to use at the national level. Using French data, [1] use local variations in APA eligibility rules to instrument formal care use. Their outcome variable was mental health. Their instrumental variable, however, cannot be used when studying the impact of formal care on informal care: it has been shown that departments often take informal care provided by relatives into account when deciding on individuals' eligibility [2].[4] More generally, any variable that depends on departmental practices in applying the APA policy is likely to be related to informal care directly, not just through its effect on formal care consumption.

To find an instrument for formal care, we investigated potential individual determinants explaining differences in the amount consumed. Socio-demographic variables and health characteristics are not good candidates because they directly affect informal care. Proximity to the head office of a professional caregiver is one possible exogenous explanation for variations in formal care consumption. The service we consider is specific however: providers cover a given geographical area, but the location of their head office location provides little information on this area. This explains why we observe no empirical correlation between amounts of care used and proximity to providers' head office.

We also considered, on the supply side, the potential effect of the capacity of formal care providers. Individual consumption could theoretically be influenced by the capacity of formal care providers in the department. The greater the capacity, the more individuals are likely to be able to consume without restriction on the supply side. Here though there is a lack of data: the number of providers in each department is known, but not their capacity (how many beneficiaries they can serve).

In this paper, we use information on the lowest regulated price in the department from the *SolvAPA* survey. This survey reports three types of regulated prices for formal care: the lowest, the highest and the price of the largest regulated provider in the department.

[4] The APA policy is supposedly "care-blind": APA eligibility should be assessed independently of any informal care received. Field studies, however, have shown that it actually depends on departmental practices.

We chose the lowest price as an instrument for formal care since it gives information on the financial accessibility of formal care. The price of the largest provider could have been a good instrument for formal care, since it corresponds to the price for the majority of formal care users. However, there were more missing values for this variable (15 missing values vs 9 for the lowest regulated price) and its variance was lower. At the individual level moreover, it was found not to be correlated with formal care consumption, whatever the sample considered (see Table D2).

Table D2: Impact of the largest provider's price on formal care use

	All<br>Regression<br>$ln(FC)$
Biggest regulated price (log)	-0.578
	(0.433)
F-test	1.79
R^2	0.215
Individual controls	Yes
Departmental controls	Yes
Clusters	70
N	2,479

Notes: * $p < 0.10$, ** $p < 0.05$, *** $p < 0.01$. Standard errors in parentheses, clustered at the departmental level. Individuals and departmental characteristics are controlled for.
Source: CARE survey [6].

E Additional estimations and robustness checks

E.1 Using lagged values for the regulated price

We extracted the value of the regulated price in 2012 from a departmental survey conducted in that year. The "*Territoire*" survey [8] was carried out by a team of sociologists and economists to evaluate the latitude of departments in implementing the APA policy. The results show that there is considerable variability in how the policy is applied [2, 7]. They were used to construct a questionnaire that was sent to the councils of the 96 metropolitan departments in 2012.[5] 71 departmental councils responded, which is similar to the number of responses to the SolvAPA survey.

This departmental survey allows us to reproduce our main estimations using a lagged value for the regulated price. Given that the departments that responded to the survey in 2012 are not all the same as those that responded in 2015, the lagged value makes it possible to test whether our results are robust to an alternative sample (individuals consuming formal care and living in a department that responded to the 2012 survey). Table E1 presents the main characteristics of the regulated prices in 2012 and in 2015. The price distributions are similar, with a correlation coefficient of 0.88.

Table E2 presents results for our main analysis but using regulated prices from 2012 rather than 2015. In the first stage of the first part and of the second, the 2012 regulated price is correlated with the number of formal care hours consumed by individuals. The F statistic is higher than in our baseline estimations, suggesting that there are no weak instrument problems with the lagged value. At the extensive margin, an increase in formal care consumption is associated with a 0.199 percentage point decrease in the probability of consuming informal care. At the intensive margin, the association is positive but not significant at conventional levels. In summary therefore, our results are robust to this robustness check.

[5]The questionnaire is available here (in French): http://modapa.cnrs.fr/questionnaire.pdf.

Table E1: Distribution of the lowest regulated prices in 2012 and 2015

	N	Mean	Standard deviation	Min	Max
Regulated price - 2012	2,162	18.5	1.71	12.3	21.08
Regulated price - 2015	2,648	19.6	1.70	12.3	21.98

Source: Territoire [8]; SolvAPA [5].

Table E2: Estimations using departmental regulated prices in 2012

	**First part (All)**		**Second part (Informal care consumers)**	
	(1)	(2)	(3)	(4)
	Regression	IV-Probit	Regression	IV-regression
	$ln(FC)$	$Pr(IC > 0)$ Marg. Eff.	$ln(FC \mid IC > 0)$	$ln(IC \mid IC > 0)$
Regulated price in 2012 (log)	-1.081***		-1.175***	
	(0.236)		(0.289)	
Formal care hours (log)		-0.199**		0.963
		(0.078)		(.586)
F-test	21.06	-	16.47	-
R^2	0.24	-	0.25	-
Individual controls	Yes		Yes	
Departmental controls	Yes		Yes	
Clusters	64		62	
N	1,208		1,498	

Interpretation: In the first stage of the first part (resp. second part), a 1% increase in the 2012 regulated price is associated with a 1.081% (resp. 1.175%) decrease in the number of hours of formal care consumed per week.
Notes: * $p < 0.10$, ** $p < 0.05$, *** $p < 0.01$. Standard errors in parentheses, clustered at the departmental level. Individuals and departmental characteristics are controlled for. The regulated price is the lowest regulated price available in the department in 2012. Models of Equations ??, ??, ??, ??. Marg. Eff., marginal effect.
Source: CARE survey [6].

E.2 Models for weak instruments

Our instrument, while being the most relevant one we were able to find, is not sufficiently correlated with formal care consumption to avoid weak instrument problems. The first issue problem with a weak instrument is precision: a weak correlation between the instrument and the independent variable of interest can substantially reduce the precision of the estimations. The second problem lies in the bias that it might create with a limited sample size. This is a major concern here given the relatively low number of observations in our sample. Our F statistic is above the critical values defined by [12] for 15% maximal IV size

bias but is below the threshold for the 10% level.[6]

In this section, we run tests and calculate confidence intervals robust to weak instruments. Following the guidelines proposed by Cameron & Triverdi [3], we calculate confidence intervals based on the conditional likelihood-ratio (CLR) statistic, proposed by Moreira [11].[7]

The intuition is the following. In the presence of a weak instrument, the normal approximation of the t-statistic used to calculate confidence intervals is inaccurate. [11] proposes a procedure for testing the hypothesis $H_0 : \beta = \beta_0$ with weak instruments. This test is based on critical values that are functions of the data. A confidence region for the parameter that is robust to weak instruments can then be constructed by inverting the test [9].

The robust confidence interval obtained is only comparable to a confidence interval with classical IV-estimation: we therefore first perform an IV-regression for the first part (probability of consuming informal care) and the second part (amount of informal care received) of our two-part model, and then also calculate confidence intervals with the CLR test. The comparison of IV-regression models and confidence intervals based on the CLR provides insight into the importance of the bias due to the weakness of the instrument. However, these results cannot be directly compared to our baseline results, since clusters cannot be included. These estimations are based on the strong assumption of homoscedasticity and should therefore be regarded cautiously.

According to Table E3, for the first part of our model, both the 2SLS estimation and the CLR confidence interval indicate that an increase in formal care use has a significant and negative effect on the probability of receiving informal care. The results for the second part of the model are more ambiguous: while the confidence interval obtained with the 2SLS estimation includes zero, the CLR confidence interval does not. With the CLR approach, an increase in formal care use is found to have a significant and positive effect on informal

[6]We use the thresholds provided by the Stata command *ivreg2*: 16.38 for a 10% maximal IV size, 8.96 for a 15% maximal IV size, 6.66 for a 20% maximal IV size and 5.53 for a 25% maximal IV size.

[7]We used the Stata command CONDIVREG.

care use at the intensive margin. Our interpretation of results at the intensive margin of informal care should therefore be particularly cautious, since the bias induced by the weakness of our instrument seems to affect our baseline results.

Table E3: Confidence intervals robust to weak instruments

	**All**	**Informal care consumers**
Dependent variable	$Pr(IC > 0)$	$ln(IC \mid IC > 0)$
2SLS	[-0.923 ; -0.140]	[-0.032 ; 3.232]
CLR	[-1.207, -0.225]	[0.441 ; 6.662]
N	2,648	1,498

Reading: Estimations of a two-stage least squares IV model with standard confidence intervals ("2SLS") and of a conditional confidence interval from the conditional likelihood ratio ("CRL").
Notes: Individual and departmental characteristics are controlled for.
Source: CARE survey [6].

E.3 Alternative specification

Impact at the extensive margin using 2SLS As a robustness check, we compute the impact of formal care on the probability of declaring informal care using a 2SLS estimator, as described in the following equation.

$$log(FC_i) = \pi_0 + \pi_1 T_{d(i)} + \pi_2 X_i + \pi_3 Y_{d(i)} + u_i \tag{10}$$

$$\mathbf{1}_{IC_i>0|_{FC_i,X_i,Y_d(i)}} = \alpha_0 + \alpha_1 \widehat{log(FC_i)} + \alpha_2 X_i + \alpha_3 Y_{d(i)} + \varepsilon_i \tag{11}$$

Table E4 shows that according to the 2SLS estimate, a one log hour increase in formal care use is associated with a 53 percentage point decrease in the probability of declaring informal care, significant at the 10% level. The standard errors are large and therefore this result is not significantly different from the marginal effect estimated using probit analysis.

Table E4: Effect of formal care on the probability of declaring informal care estimated using 2SLS regression

	Main sample	APA beneficiaries	Living alone
Formal care hours (log)	-0.532*	-0.260*	-0.415**
	(0.306)	(0.150)	(0.198)
Observations	2,648	1,172	1,881

Interpretation: This Table shows the results in the main sample, sample of APA beneficiaries and sample of over-60s living alone, using a 2SLS estimator in a linear model. An increase of one-log hour in formal care use is associated with a 53 percentage point decrease in the probability of declaring informal care, significant at the 10% level.
Notes: Standard errors in parentheses, clustered at the departmental level. * $p < 0.10$, ** $p < 0.05$, *** $p < 0.01$. The log-number of formal care hours is instrumented by the lowest regulated price in the department. Individual and departmental characteristics are controlled for.
Source: CARE survey [6].

E.4 Dealing with missed caregivers

The variable we use is built on respondents' statements: recipients of informal care are defined as respondents who declared informal caregivers and quantified the amount of care they provided. This definition is restrictive: for some informal caregivers, the recipient was not able to quantify the care they provided and these carers were therefore not taken into account. They could be called "missed caregivers". Three quarters of respondents (73.6%) had no missed caregivers.

Among the remaining respondents, 9.2% listed both quantified and non-quantified informal caregivers and are therefore identified as care recipients but with an underestimated amount of informal care received. The final 17.2% had only missed caregivers and were therefore identified as not receiving informal care even though they had declared receiving some.

Table E5 presents the determinants of having at least one missed caregiver. Younger respondents and those living alone were less likely to be unable to quantify the amount of care provided by any caregiver. Surprisingly, the association with disability status is non-linear: the most severely disabled (disability group 1/2) and less disabled individuals (disability groups 5/6) were significantly less likely to have at least one missed caregiver.

We consider an alternative definition of informal care recipients including all recipients who declare a caregiver – regardless of whether the amount of care was quantified. Results for the first part of our two-part model with this alternative definition (Table E6) are consistent with our baseline results: an increase in the amount of formal care consumption is associated with a decrease in the probability of receiving informal care, with a similar effect size.

Table E7 presents the results of estimates excluding individuals with at least one missed caregiver, showing that our baseline results are robust to this exclusion. Note that the probability of having at least one missed caregiver is not correlated with the number of formal care hours consumed or with our instrument (probit model analysis controlling for parents' characteristics).

Table E5: Probability of having at least one missed caregiver

	Has at least one missed caregiver
Woman	-0.0292
	(0.0686)
Age	0.00191
	(0.00348)
Lives alone	-0.153**
	(0.0645)
Disability group	-0.0411*
	(0.0211)
Has the *baccalauréat*	-0.00233
	(0.0862)
Has children	0.269***
	(0.0889)
Income (/1000)	-0.00875***
	(0.00316)
Proxy	0.131**
	(0.0627)
Observations	2,648

Notes: * $p < 0.10$, ** $p < 0.05$, *** $p < 0.01$. Standard errors in parentheses. Estimation of a probit model.
Source: CARE survey [6].

Table E6: Alternative definition of informal care use

	Probability to receive some informal care Marg. Eff.
Formal care hours (log)	-0.230***
	(0.072)
Individual controls	Yes
Departemental controls	Yes
N	2,648

Interpretation: An exogenous increase of 1% in the amount of formal care consumed is associated with a significant 23.0 percentage point decrease in the probability of declaring receiving informal care. The sample for this model includes respondents who declared receiving informal care but did not quantify how much care was received.
Notes: * $p < 0.10$, ** $p < 0.05$, *** $p < 0.01$. Standard errors in parentheses. Estimations of an IV Probit model where the log-number of formal care hours is instrumented by the lowest regulated price in the department. Individual and departmental characteristics are controlled for. Marg. Eff., marginal effects.
Source: CARE survey [6].

Table E7: Results for the subsample of respondents with no missed caregivers

	**First part (All)**		**Second part (Informal care consumers)**	
	Regression $ln(FC)$	IV-Probit $Pr(IC > 0)$ Marg. Eff.	Regression $ln(FC\|IC > 0)$	IV-regression $ln(IC\|IC > 0)$
Regulated price (log)	-0.913***		-0.821***	
	(0.243)		(0.267)	
Formal care hours (log)		-0.261***		1.083
		(0.066)		(0.695)
F-test	14.08	-	7.52	-
R^2	0.28	-	0.25	-
Individual controls	Yes		Yes	
Departmental controls	Yes		Yes	
Clusters	75		74	
N	1,949		1,255	

Interpretation: In the first stage of the first part (resp. second part), a 1% increase in the regulated price in the department is associated with an average decrease of 0.913% (resp. 0.821%) in the number of hours of formal care used per week. An increase of one log-hour in the amount of formal care consumed is associated with a 26.1 percentage point decrease in the probability of receiving informal care. Among informal care recipients, an exogenous increase of one log-hour in the amount of formal care consumed is associated with a non-significant decrease in the amount of informal care received.
Notes: * $p < 0.10$, ** $p < 0.05$, *** $p < 0.01$. Standard errors in parentheses, clustered at the departmental level. Individuals and departmental characteristics are controlled for. The regulated price is the lowest regulated price available in the department. Marg. Eff., marginal effects.
Source: CARE survey [6].

References

[1] T. Barnay and S. Juin. Does home care for dependent elderly people improve their mental health? *Journal of Health Economics*, 45:149–160, 2016.

[2] S. Billaud, C. Bourreau-Dubois, A. Gramain, H. Lim, F. Weber, and J. Xing. La prise en charge de la dépendance des personnes âgées: les dimensions territoriales de l'action publique. Rapport final réalisé pour la MiRe - DREES, 2012.

[3] A. C. Cameron and K. P. Trivedi. *Microeconometrics using Stata*. Stata Press, College Station, TX, 2009.

[4] L. Carrino, C. E. Orso, and G. Pasini. Demand of long-term care and benefit eligibility across European countries. *Health Economics*, 27(8):1175–1188, Aug. 2018.

[5] DREES. *Base solvAPA sur le fonctionnement et les tarifs de l'APA à domicile en 2015*. 2015.

[6] DREES. *Enquête Capacités, aides et ressources des seniors - volet Ménages*. 2015.

[7] A. Gramain and J. Xing. Tarification publique et normalisation des processus de production dans le secteur de l'aide à domicile pour les personnes âgées. *Revue française des affaires sociales*, (2012/2-3):218–243, 2012.

[8] LEDa-LEGOS and CES. *Enquête Territoire - APA et tarification des services d'aide à domicile*. Agnès Gramain and Jérôme Wittwer (dir.), 2012.

[9] A. Mikusheva. Robust confidence sets in the presence of weak instruments. *Journal of Econometrics*, 157(2):236–247, 2010.

[10] P. A. Moran. The interpretation of statistical maps. *Journal of the Royal Statistical Society. Series B (Methodological)*, 10(2):243–251, 1948.

[11] M. J. Moreira. A conditional likelihood ratio test for structural models. *Econometrica*, 71(4):1027–1048, 2003.

[12] J. H. Stock and M. Yogo. Testing for Weak Instruments in Linear IV Regression. In *Identification and Inference for Econometric Models: Essays in Honor of Thomas Rothenberg.*, pages 81–108. Cambridge University Press, Cambridge, 2005.
